# Overview of Cytogenetics in the Subtribe Orchidinae (Orchidaceae) in the Mediterranean Region

**DOI:** 10.3390/plants15091361

**Published:** 2026-04-29

**Authors:** Alessio Turco, Robert Philipp Wagensommer, Antonella Albano, Pietro Medagli, Enrico Vito Perrino, Saverio D’Emerico

**Affiliations:** 1 Faculty of Education, Free University of Bozen-Bolzano, 39042 Brixen-Bressanone, Italy; alessio.turco@unibz.it; 2 Department of Biological and Environmental Sciences and Technologies, University of the Salento, 73100 Lecce, Italy; antonella.albano@unisalento.it (A.A.); pietro.medagli@unisalento.it (P.M.); 3 Department of Agriculture, Food, Natural Resources and Engineering, University of Foggia, 71100 Foggia, Italy; enrico.perrino@unifg.it; 4 “Aldo Moro” University of Bari, 70125 Bari, Italy; sdeme@yahoo.it

**Keywords:** *Anacamptis* s.l., *Chamorchis*, chromosome numbers, classical and molecular cytogenetics, *Dactylorhiza* s.l., evolution, *Gymnadenia* s.l., karyomorphological comparison, *Neotinea* s.l., *Ophrys*, *Orchis* s.s., *Platanthera*, *Serapias*

## Abstract

In the Mediterranean region, for over 150 species belonging to the subtribe Orchidinae, chromosome number has been documented and found to range from 2n = 32 to 2n = 42. This work renews and updates chromosome numbers and reports a karyomorphological comparison between species with 32 or 36 chromosomes and species with 40 or 42 chromosomes. Notably, in the 32,36-chromosome group, species within the genus *Anacamptis* s.l. show substantially similar karyomorphology, although some species exhibit differences in chromosome structure and heterochromatin distribution. In contrast, in the 40,42-chromosome group, the chromosomes are comparatively smaller and therefore difficult to sort into karyotypes when standard staining techniques are used. However, the two groups display distinct heterochromatin patterns, particularly in centromeric and telomeric regions. Given the growing body of information in this field, a review of current cytogenetic knowledge is warranted. In this report, the authors present classical and molecular cytogenetic data and highlight important aspects of karyotypic evolution in Orchidinae. Traditional karyotypic analysis, based on stained mitotic chromosomes, can be used to distinguish and identify taxonomic groups. Karyomorphometric studies in particular reveal subtle differences between closely related chromosome sets within Orchidinae. Furthermore, wide variation among genera in terms of heterochromatin content was observed. Further comparative data between the two above-mentioned groups are summarized.

## 1. Introduction

European orchids, renowned for their remarkable morphological and ecological diversity [1,2], provide an excellent model for investigating evolutionary mechanisms in plants. The subtribe Orchidinae, which comprises numerous genera, has attracted considerable scientific interest and has yielded important insights into morphology, chromosome numbers, karyomorphology, structural variation, and molecular evolution [3,4,5,6,7,8,9,10,11,12,13,14,15,16,17,18,19,20].

Chromosome research, central to understanding heredity and evolution, has long provided essential insights into biodiversity, adaptation and evolutionary processes [21,22]. The Orchidinae, comprising mainly terrestrial species, are particularly suitable for cytogenetic investigation due to their ecological specificity and diverse reproductive strategies [4,23,24]. Cytogenetic studies have revealed considerable variation in chromosome numbers: most species are diploid, while others exhibit aneuploidy or polyploidy [5,25]. Chromosome analyses have also highlighted karyotypic asymmetry and structural rearrangements that may drive speciation and adaptive radiation within the group [26]. Furthermore, studies of meiotic behavior provide evidence of hybridization—including areas where this is frequently observed—in several genera, including *Anacamptis*, *Ophrys* and *Orchis* [5,25,27,28].

Classical cytogenetic approaches, including chromosome counts from mitotic and meiotic preparations, have been fundamental to these discoveries [5,19,25,28]. Comparative genomic analyses across orchid species allow researchers to identify genome regions that have undergone major evolutionary change [5,6,9,10,19,20,23,27,28]. In addition, meiotic analysis offers insights into chromosome pairing, recombination and gamete formation [29,30]. Studies of DNA methylation and histone modifications shed further light on how environmental factors influence gene expression and contribute to phenotypic variation [19,20,31]. Advances in molecular cytogenetics, such as fluorescence in situ hybridization (FISH) and genomic in situ hybridization (GISH), have deepened our understanding by enabling the localization on chromosomes of specific DNA sequences, including ribosomal DNA loci and telomeric regions [19,20,28,32,33,34,35]. These techniques have greatly improved our knowledge of chromosomal evolution and genome organization within the Orchidinae.

This overview of cytogenetic findings on the Orchidinae of the Mediterranean region places particular emphasis on chromosome number variation, morphology and methodological advances. Nonetheless, many questions remain regarding the role of cytogenetic mechanisms in environmental adaptation and pollinator interactions. By drawing together current knowledge, we aim to ascertain the evolutionary dynamics shaping the cytogenetic diversity of European orchids and to highlight their broader significance for taxonomy, phylogeny and conservation.

## 2. Chromosome Number Variation in Orchidinae

**Figure 1:**
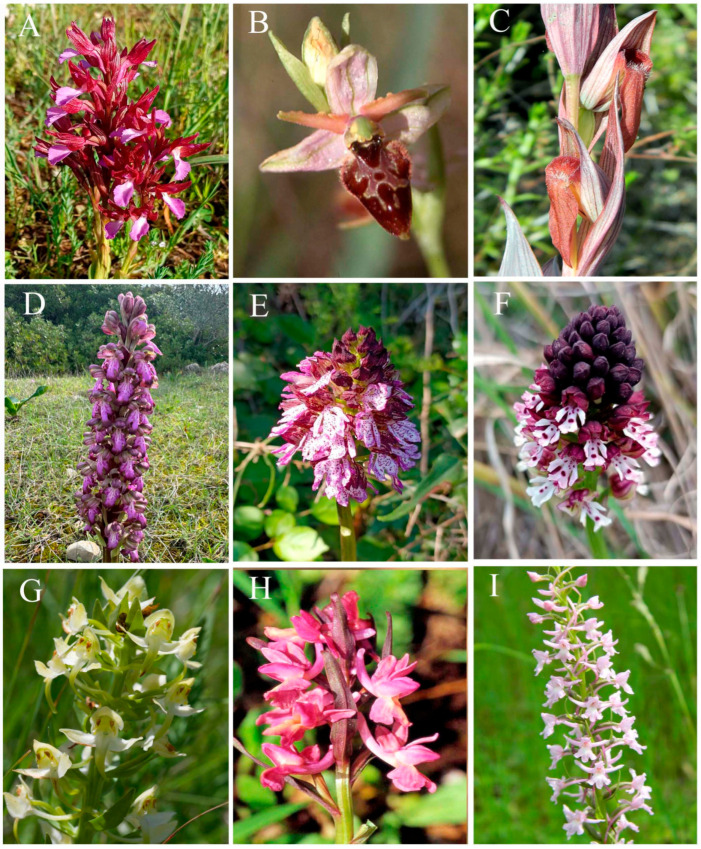
Representative taxa from the Mediterranean region: (**A**) *Anacamptis papilionacea* (L.) R.M.Bateman, Pridgeon & M.W.Chase; (**B**) *Ophrys mateolana* Medagli, D’Emerico, Bianco & Ruggiero; (**C**) *Serapias vomeracea* (Burm.f.) Briq.; (**D**) *Himantoglossum robertianum* (Loisel.) P.Delforge; (**E**) *Orchis purpurea* Huds.; (**F**) *Neotinea ustulata* (L.) R.M.Bateman, Pridgeon & M.W.Chase; (**G**) *Platanthera chlorantha* (Custer) Rchb.; (**H**) *Dactylorhiza romana* (Sebast.) Soò; (**I**) *Gymnadenia conopsea* (L.) R.Br.

**Figure 2:**
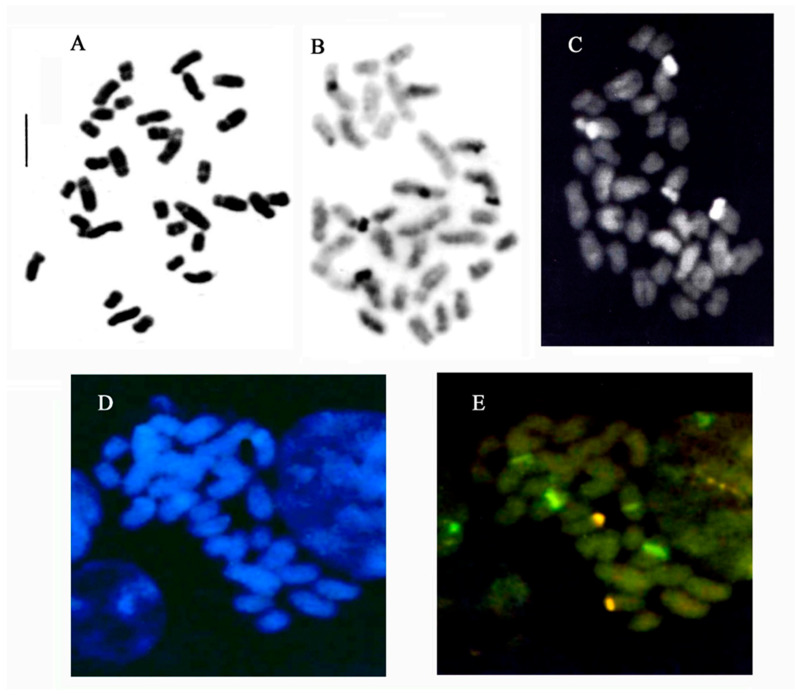
Some techniques used applied to *Anacamptis papilionacea* (2n = 2x = 32): (**A**) Feulgen staining; (**B**) Banding with Giemsa; (**C**) Banding with fluorochrome Hoechst 33258; (**D**) Banding with fluorochrome DAPI; (**E**) Fluorescence in Situ Hybridization (FISH) in orange 35S rDNA and green 5S rDNA (adapted from Refs. [19,28,33]). Scale bar = 5 µm.

**Figure 3:**
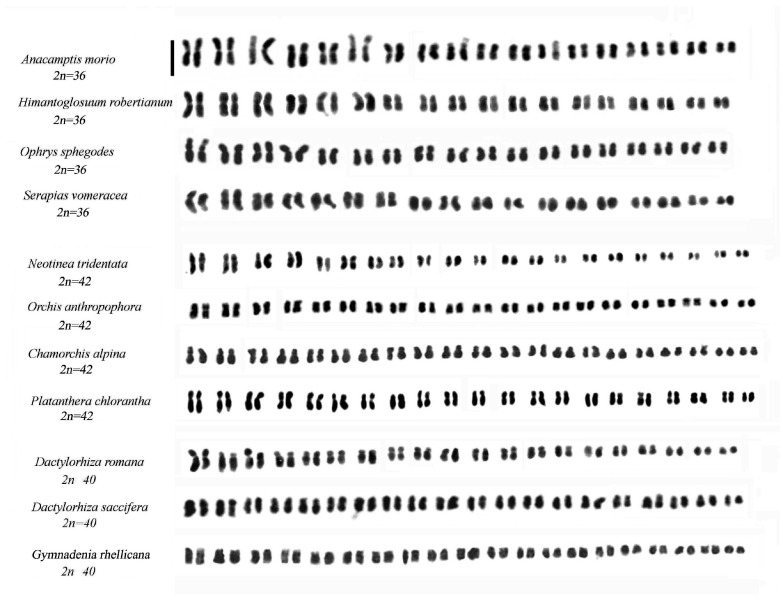
Karyotypes of selected genera of the Orchidinae subtribe (adapted from Refs. [19,20]). Scale bar = 5 µm.

Figure 1 shows representative species from the genera *Anacamptis* s.l., *Dactylorhiza*, *Gymnadenia*, *Himantoglossum*, *Neotinea* s.l., *Ophrys*, *Orchis* s.s., *Platanthera* and *Serapias*. The nomenclature used for the species follows GIROS [36], Kreutz [2], and in some cases POWO [37]. Figure 2 and Figure 3 show representative karyomorphologies of chromosome complements in selected genera.

### Chromosome Numbers

The subtribe Orchidinae comprises genera and species with diverse chromosome numbers and ploidy levels. In the Mediterranean region, five basic chromosome numbers, x = 16, 17, 18, 20 and 21, have been identified across 152 species, 17 of which display both aneuploid and diploid or polyploid counts. In many species, individual chromosomes can be distinguished using standard karyotyping or banding techniques (Figure 2); however, this process becomes challenging in taxa with small chromosomes, such as *Dactylorhiza*, *Gymnadenia* s.l., *Neotinea* s.l. and *Orchis* s.s. [20,38].

The present study draws on an extensive dataset of chromosome records for genera and species belonging to the Orchidinae of the Mediterranean region. The most recent comprehensive compilation of chromosome numbers in this subtribe was published by D’Emerico [12], and since then a substantial amount of new data has become available. These additional records have greatly enriched our cytological understanding of this important plant group.

The data from our present overview of the literature are presented in Table 1, Table 2, Table 3 and Table 4. Table 1 reports updated chromosome numbers for several genera and species based on recent and reliable data.

All the Orchidinae genera found in the Mediterranean region are represented in Table 2. Diploid chromosome numbers range from 2n = 32 to 2n = 42, while polyploid and aneuploid taxa have higher values. Another notable feature is that most genera are characterized by a single basic chromosome number.

Table 3 summarizes the results obtained for selected species within the 32,36- and 40,42-chromosome groups, while the main cytogenetic comparisons between 32,36-chromosome and 40,42-chromosome orchid groups are presented in Table 4.

## 3. Karyomorphology

The genus *Gennaria*, which includes only the species *Gennaria diphylla* (Link) Parl., is characterized by a chromosome number of 2n = 34 [61]. Four genera, *Anacamptis*, *Himantoglossum*, *Ophrys* and *Serapias*, comprising numerous species, all have a chromosome number of 2n = 2x = 36 (Figure 3). These genera provide a large amount of data, including genomic characteristics that facilitate their study, such as the low chromosome number and adequate chromosome size, the latter being particularly useful for assessing karyotype symmetry.

The genus *Anacamptis* includes many species with the diploid chromosome number 2n = 2x = 36. Tetraploid cytotypes (2n = 4x = 72) have been reported, along with cytotypes possessing 2n = 3x = 54 chromosomes [19,25,38,43,44,45,46,57,93]. Rare cases of triploidy (2n = 3x = 54) have been observed in *A. laxiflora*, *A. fragrans* and *A. pyramidalis*. Meiotic plates at metaphase I examined by EMC revealed numerous trivalents, confirming the autopolyploid origin of the above-mentioned specimens [25,38,45]. Detailed karyological analyses have revealed a consistent “basic karyotype” across all studied *Anacamptis* species [5,19,41,42,45]. One feature of this is that in addition to chromosome pairs bearing secondary constrictions on the short arm, secondary constrictions may also occur on the long arm. It is also possible to observe chromosomes marked by a secondary constriction on the short arm and a microsatellite on the long arm. *Anacamptis papilionacea* (the only species with 2n = 2x = 32), *A. coriophora* and *A. fragrans* (the latter two both with 2n = 2x = 36) all exhibit a notably asymmetrical karyotype dominated by submetacentric chromosomes. Within the *Anacamptis* genus, *A. pyramidalis* showed three levels of ploidy, diploids (2n = 2x = 36), triploids (2n = 3x = 54) and tetraploids (2n = 4x = 72), often associated with morphological and chromatic differences [25,45,94]. In this species, cytotypes differ in geographical distribution, with diploids occupying restricted areas and undisturbed vegetation, while tetraploids are more widespread (being found for example on uncultivated land). Triploid individuals are usually found in geographically identical areas, where both the other two cytotypes are present, and arise from cross-breeding between diploid and tetraploid individuals [45].

In *Anacamptis*, therefore, polyploidy is prevalent, with recurring diploid-triploid-tetraploid series and clear evidence of autopolyploid origin (e.g., trivalent formation during meiosis) [25,45,94]. Cytotypes are not randomly distributed but are correlated with ecology and geography, with diploids occupying more restricted, stable habitats and polyploids exhibiting a broader ecological range [45]. This pattern suggests that polyploidy enhances ecological flexibility and may facilitate range expansion.

The genus *Himantoglossum* C.Koch is reported to have a diploid chromosome number of 2n = 2x = 36 and includes three species: *H. robertianum*, *H. hircinum* and *H. adriaticum*. However, cases of aneuploidy (2n = 36 + 1B) have been documented in both *H. hircinum* and *H. adriaticum* [43,45]. The three species show similar karyotype structures, characterized predominantly by metacentric chromosomes [41,45]. *Himantoglossum* contrasts with *Anacamptis* in maintaining both numerical and structural stability, with only minor deviations (e.g., B chromosomes). The genus *Ophrys* L. is characterized by a stable diploid chromosome number of 2n = 2x = 36 [19,72,74]. However, cases of polyploidy have been reported within the *O. fusca*–*O. lutea*–*O. omegaifera* complex, with French and Iberian populations exhibiting chromosome numbers of 2n = 4x = 72 or 2n = 5x = 90 [73]. In contrast, Italian populations of *Ophrys* are consistently diploid, with the exception of *O. lupercalis* Devillers-Terschuren and Devillers, which displays a chromosome number of 2n = 4x = 72 in the Gargano Promontory [75]. Additionally, an autotriploid individual of *O. tenthredinifera* with 2n = 3x = 54 has been recorded, and some cases of somatic aneuploidy with 2n = 37, 38 or 39 chromosomes have also been documented [25,59,72,80,95]. However, the presence of aneuploidy in this group appears to be linked to hybridization and introgression. Nevertheless, supernumerary chromosomes in both somatic metaphases and meiotic cells have only been observed in *O. bertolonii* × *O. tarentina*, *O. apulica* × *O. bombyliflora*, *O. biscutella* and *O. promontorii* [25,72]. The “basic karyotype” of *Ophrys* typically includes three pairs of chromosomes bearing satellites, the first of which is distinguished by a large secondary constriction on the short arm of a metacentric or submetacentric chromosome. Pairs exhibiting secondary constrictions in the *Ophrys* genus have only been observed using the traditional Feulgen technique. In *O. tenthredinifera* s.l., the only species analyzed with the FISH technique, it was not possible to identify the chromosome pair carrying the 35S rDNA cluster. Considerable variation in chromosome morphology and the size of the secondary constriction has been reported within the genus [19,25,74,79]. Karyotype differentiation within *Ophrys* can be assessed by various asymmetry indices [96,97,98]. The taxa form a continuum from more symmetrical karyotypes, characterized by predominantly metacentric chromosomes, to more asymmetrical ones with a higher proportion of submetacentric chromosomes. Highly symmetrical karyotypes occur in *Ophrys bombyliflora*, the *O. fusca*–*O. lutea*–*O. omegaifera* complex and *O. tenthredinifera*, whereas *O. insectifera* and *O. bertolonii* exhibit a greater number of submetacentric chromosomes [33,74].

A similar pattern to that observed in *Anacamptis*, albeit less pronounced, is also found in *Ophrys*. In this genus, diploidy predominates, while instances of localized polyploidy and aneuploidy are reported in association with hybridization and introgression, particularly among closely related species groups [25,72]. Karyotypic variation in *Ophrys* is better interpreted as a continuum of symmetry in terms of features such as the relative distribution of chromosome sizes and centromere positions rather than as a set of discrete categories [33,74,96,97,98]. This variability probably reflects gradual chromosomal restructuring and may contribute to speciation processes, for example by promoting reproductive isolation or meiotic instability.

The genus *Serapias* comprises a group of entities that are often difficult to identify taxonomically and are distributed across the Mediterranean basin, the Canary Islands, and the Azores. Most investigated species have a chromosome number of 2n = 2x = 36 [42,54,57,88,93,99,100,101]. Exceptions include *S. gregaria*, *S. lingua*, *S. olbia* and *S. strictiflora*, which possess a polyploid number of 2n = 4x = 72 [89]. Triploid complements (2n = 3x = 54) have been recorded in *S. intermedia* subsp. *hyblaea* Cristaudo, Galesi and R. Lorenz, *S. todaroi* Tineo (*S. lingua* × *S. parviflora*) and *Serapias* × *lupiensis* (*S. lingua* × *S. politisii*) [101,102]. The karyotypes are moderately asymmetrical, with a predominance of submetacentric and subtelocentric chromosomes [88]. *Serapias* thus occupies an intermediate position: it is numerically stable but taxonomically complex, with polyploidy and hybrid triploids contributing to its poorly resolved species boundaries [101,102]. Its moderately asymmetrical karyotypes constitute further evidence of this transitional character.

In the 42-chromosome group (e.g., *Neotinea* s.l., *Orchis* s.s.), the chromosomes are comparatively small, and karyotypes are therefore difficult to distinguish using standard staining techniques. Cytological studies of *Neotinea* Rchb. have revealed a basic chromosome number of x = 21 and predominantly diploid species [20]. The only exception is *N. commutata*, which is tetraploid with 2n = 4x = 84 [20,69]. The species *N. lactea*, *N. tridentata* and *N. ustulata* have also shown 2n = 42 + 1B [20,45]. Comparative karyotype studies between the 32,36-chromosome *Anacamptis* group and the 42-chromosome *Neotinea* group show that the latter differs in having smaller chromosomes and a more complex karyomorphology. Furthermore, the karyotypes of *Neotinea* species consist mainly of metacentric chromosomes.

Cytological studies in *Orchis* s.s. have revealed a diploid chromosome number of 2n = 2x = 42 for all species except *O. patens*, which is tetraploid, with 2n = 4x = 84 [85]. A rare triploid case has been reported for *O. italica*, with 2n = 3x = 63 [25]. Notably, the karyomorphology of this group is very similar to that of *Neotinea*, especially the presence of smaller-sized chromosomes [19,20]. Within *Orchis* s.s. the *O. mascula* complex has long been the subject of taxonomic debate [15,103]. In this context, the karyomorphological comparison of *O. provincialis* and *O. mascula*, both belonging to the *O. mascula* subgroup, is particularly informative, as the two species are morphologically similar and closely related phylogenetically [27]. Thus, the karyological similarity between *Neotinea* and *Orchis* s.s. supports a close relationship but also highlights the limited discriminatory power of chromosomal morphology, meaning that taxonomic approaches need to be combined with further cytogenetic data [19,20,27].

*Dactylorhiza* Neck. ex Nevski, with a basic chromosome number of x = 20, includes diploid (2n = 2x = 40) and polyploid (2n = 4x = 80, 2n = 6x = 120) species [54,104]. Triploids (2n = 3x = 60) are reported for *Dactylorhiza insularis* and *D. maculata* subsp. *meyeri* [42,44,105]. Cytological analyses of *D. romana* from southern Italy have confirmed 2n = 40, and some aneuploid individuals with 2n = 40 + 1 − 3B have also been observed. The chromosomes in *Dactylorhiza* are small, like those of the 42-chromosome *Neotinea* and *Orchis* groups. Among the numerous species in this genus, only *D. insularis* [42], *D. maculata* subsp. *meyeri* [105] and *D. romana* [45] have been karyologically examined.

The genus *Gymnadenia* R.Br., with a basic chromosome number of x = 20, also includes diploid cytotypes with 2n = 2x = 40, such as *G. carpatica*, *G. rhellicani*, *G. gabasiana*, *G. corneliana* and *G. lithopolitanica* [62,68], and polyploid cytotypes with 2n = 4x = 80 and 2n = 6x = 120, such as *G. nigra*, *G. miniata*, *G. widderi*, *G. stiriaca*, *G. archiducis-joannis*, *G. dolomitensis* and *G. buschmanniae* [48,63,66,68,106,107]. These chromosome numbers indicate that polyploidy has played a central role in the evolution of the genus. The chromosomes are small, and the karyotype morphology is highly complex; thus, only Giemsa C-banding provides sufficient detail for karyomorphological interpretation. Together with *Dactylorhiza*, *Gymnadenia* may be said to represent a third evolutionary pathway characterized by extensive and recurrent polyploid radiation [54,104]. In these taxa, polyploidy is not exceptional but a key feature, underpinning diversification.

In the genus *Platanthera* Rich., the chromosome number observed is 2n = 42. The chromosomes of the few species examined [87] are larger than those of most *Neotinea*, *Orchis*, *Dactylorhiza* and *Gymnadenia* species. *Platanthera chlorantha* and *P. algeriensis* display a symmetrical karyotype.

In *Chamorchis* Rich., the only species *C. alpina* (2n = 42) exhibits a moderately asymmetrical karyotype. The chromosome size and karyomorphology of *C. alpina* differ considerably from those of other genera with 42 chromosomes (e.g., *Orchis* s.s., *Neotinea* s.l., *Traunsteinera*). Indeed, in *C. alpina*, the centromere positions are clearly distinguishable in all chromosomes [20,48]. In *Traunsteinera*, the species *T. globosa* possesses 2n = 42 and its chromosomes are small, resembling those of the 42-chromosome *Orchis* s.s. and *Neotinea* s.l. groups [38].

In the genus *Pseudorchis*, the species *P. albida* has a chromosome number of 2n = 42, with chromosome sizes ranging from 3 to 1.5 µm [64].

Despite sharing the same chromosome number, the genera *Platanthera*, *Chamorchis*, *Traunsteinera* and *Pseudorchis* exhibit divergent karyomorphological strategies [20,48,64,87]. These strategies range from symmetrical complements with relatively large chromosomes (in *Platanthera*) to more distinctive and diagnostically informative karyotypes (in *Chamorchis*), indicating that chromosome number alone is insufficient to infer karyological similarity.

### Gaps in Current Knowledge

Overall, three major insights emerge. First, chromosome number is conserved within major lineages but is dissociated from karyotype structure, which can vary substantially. Second, polyploidy and hybridization are key drivers of diversification, although their prevalence differs markedly among genera. Third, chromosome size and morphology strongly influence analytical resolution, with implications for both cytogenetic interpretation and taxonomic clarity.

New species of wild orchids from the Mediterranean region continue to be identified in both the 32,36- and 40,42-chromosome groups [1,2]. Consequently, not only new chromosome numbers, particularly cases of polyploidy, but also karyomorphological data need to be compiled and clarified (e.g., in *Ophrys*, *Serapias*, *Orchis* s.s., *Dactylorhiza* and *Gymnadenia*).

## 4. Evolutionary Inferences and Phylogenetic Considerations

The 32,36-chromosome orchid group, particularly *Anacamptis* s.l. and related genera, exhibits notable karyotypic diversity and some polyploidization [5,12,19,25,33,41,74,75,76,88,101], and it may represent a more conserved pattern than the 40,42-chromosome group. In this context, certain chromosomal characteristics, such as the dimensions used to calculate karyotype symmetry, heterochromatin content and the frequency of polyploidy, are particularly important for diagnosis. Cytogenetic evidence indicates that *Anacamptis* and *Himantoglossum* share conserved basic karyotypes with limited chromosomal rearrangement. In *Ophrys*, however, karyotype variation appears to have contributed to increased speciation and may explain the absence of intergeneric hybrids [28]. Within this group, polyploidization is well documented in *Anacamptis pyramidalis* and *A. berica* (2n = 54, 63, 72) [25,45,94], as are cases of allotriploidy and structural hybridization, for example *Anacamptis* × *gennarii* (2n = 34, a hybrid with parental counts of 36 and 32 chromosomes, showing 54 in some individuals) [108] and *Anacamptis collina* × *A. morio* with 2n = 54 [45]. The genus *Serapias* likewise includes both autopolyploid and allopolyploid taxa (e.g., *S. ausoniae*, *S. lingua*) [19,33]. However, cases of polyploidy remain confined to a few species belonging to the genera *Anacamptis* (e.g., *Anacamptis pyramidalis*), *Ophrys* (e.g., *O. forestieri*), and *Serapias* (e.g., *S. lingua*) [75,76,88,101].

In contrast, the 40,42-chromosome group displays more complex evolutionary pathways, with clear examples of polyploid evolution such as *Neotinea commutata* [69]. The coexistence of autopolyploid and allopolyploid events suggests adaptive responses to ecological pressures. The presence of B chromosomes and epigenetic features, for example in *Orchis mascula*, indicates ongoing genomic evolution and potential adaptive flexibility [58]. Numerous species in this group, including those of *Neotinea*, *Orchis*, *Dactylorhiza* and *Gymnadenia*, exhibit great karyotype complexity and polyploidy, indicating enhanced cytogenetic development. Polyploidization in this group is typically more structured, often with autopolyploidy followed by allopolyploidizationby integrating multiple approaches, including cytogenetic analyses (both classical and molecular), molecular analyses (e.g., nuclear and plastid DNA), identification of genetic markers, morphological studies and phylogenetic reconstructions [35,70,109,110,111,112,113]. Polyploidy is widespread in *Dactylorhiza* and *Gymnadenia*, with chromosome numbers ranging from 2n = 2x = 40 to 2n = 6x = 120. Chromosome behaviors during meiosis, such as stable bivalent formation, confirm established allopolyploidy (e.g., *Neotinea commutata*) [20].

Taken together, these differences in chromosomal architecture and polyploid dynamics provide essential insights into speciation and diversification within the subtribe Orchidinae.

### Evolutionary Lineage

For the 32,36-chromosome group, extensive karyological data are available, allowing the determination of evolutionary lineage. However, the discovery of numerous new species raises important questions regarding their speciation which cannot be exhaustively investigated using traditional cytogenetic methods. Furthermore, less is known about the 40,42-chromosome group (e.g., *Dactylorhiza* and *Orchis* s.s.), mainly due to the presence of smaller chromosomes that are more difficult to analyze.

## 5. Heterochromatin Distribution and Cytogenetic Markers in Representative Orchidinae

Numerous species within the representative Orchidinae exhibit variable heterochromatin content and distribution, the latter sometimes correlating with karyotype asymmetry (e.g., *Anacamptis papilionacea*, *A. coriophora*). The most detailed studies in this regard concern *Anacamptis* s.l., *Dactylorhiza* s.l., *Gymnadenia*, *Himantoglossum* s.l., *Ophrys*, *Orchis* s.s., *Neotinea* s.l., *Serapias* and the *Traunsteinera*–*Chamorchis* complex [19,20,33,38].

In *Anacamptis*, banding analyses have demonstrated the presence of substantial heterochromatic regions, with variation both among species and among populations. Similarly, in *Ophrys*, C-banding and CMA staining produce highly consistent patterns, with CMA^+^ regions largely co-localizing with C^+^ bands. Distinct heterochromatin distributions have been reported for species groups, such as the relatively symmetrical and heterochromatin-poor *Ophrys fusca* group and the more asymmetrical *O. bertolonii* group. In *Serapias*, C-banding reveals prominent centromeric heterochromatin across species, indicating a relatively uniform cytogenetic profile [5,19,33,74,88,101].

In contrast, taxa within the 2n = 40,42-chromosome group, including *Neotinea* s.l. and *Dactylorhiza*, show agreement between DAPI and C-banding patterns, suggesting more consistent organization of heterochromatic regions. Within *Orchis* s.s., species such as *O. italica*, *O. militaris*, *O. purpurea*, *O. quadripunctata* and *O. simia* display largely neutral responses to both C-banding and DAPI staining [20]. However, further analyses of specimens from additional populations are required in order to confirm these results. In the *Orchis mascula* group, notable differences occur: *O. provincialis* exhibits thin centromeric and telomeric bands, whereas *O. mascula* shows extensive centromeric heterochromatin that is C-positive but DAPI-negative. Additionally, DAPI staining frequently reveals bright telomeric or subtelomeric signals, indicative of AT-rich repetitive DNA [20,27]. In *Neotinea* (e.g., *N. lactea*, *N. tridentata*), DAPI^+^ regions consistently co-localize with C-bands, contrasting with the pattern observed in the *Orchis mascula* group [20,27]. In *Dactylorhiza romana*, C-banding has also enabled the identification of large B-chromosomes rich in constitutive heterochromatin. These supernumerary chromosomes are large and resemble the longest A-chromosomes in terms of morphology and size [20,58,114]. Other genera show distinct patterns: in *Chamorchis alpina*, heterochromatin can occupy most of the chromosome length, leaving euchromatin restricted to terminal regions [48], whereas *Traunsteinera globosa* exhibits only small centromeric heterochromatic bands [38].

The observed heterochromatin patterns reflect underlying differences in the composition, distribution and organization of repetitive DNA across Orchidinae genomes. Co-localization of CMA^+^ and C-bands in genera such as *Ophrys* suggests the presence of GC-rich repetitive elements within constitutive heterochromatin, whereas strong DAPI signals indicate AT-rich satellite DNA, particularly in the telomeric regions of *Orchis* species [20,74]. The contrasting relationships between DAPI staining and C-banding in different taxa highlight distinct genomic architectures. For instance, the lack of co-localization between DAPI^+^ regions and C-bands in the *Orchis mascula* group suggests the presence of heterogeneous heterochromatin types, potentially differing in sequence composition or epigenetic state [20]. Conversely, the consistent overlap observed in *Neotinea* indicates a more uniform organization of repetitive DNA [20].

Variation in heterochromatin content and distribution within genera such as *Anacamptis*, particularly between geographically separate populations (e.g., *A. papilionacea*), further suggests that repetitive DNA is dynamic and responsive to ecological and demographic factors [19,28]. The presence of heterochromatin-rich B-chromosomes in *Dactylorhiza romana* also points to the accumulation of repetitive sequences in supernumerary genomic elements [58]. Overall, these cytogenetic patterns indicate that repetitive DNA plays a central role in shaping genome structure, contributing to differences in chromosome morphology and banding profiles in closely related taxa.

The diversity of heterochromatin distribution patterns across Orchidinae provides important insights into chromosomal evolution and species divergence. In *Anacamptis*, karyological data support the existence of a conserved ancestral karyotype characterized by predominantly metacentric chromosomes and low asymmetry, as observed in *A. morio* and *A. longicornu*. Species that are descended from the same ancestral karyotype but appear to be the result of greater evolutionary transformation, such as *A. collina*, *A. papilionacea* and *A. coriophora*, exhibit increased karyotype asymmetry and higher heterochromatin content, consistent with progressive chromosomal rearrangement [5,19,33]. This trend suggests that heterochromatin expansion and structural chromosome changes have contributed to lineage diversification. Similar patterns are observed in *Serapias*, where high heterochromatin content and karyotype asymmetry, combined with low ITS sequence divergence, point to relatively recent and rapid chromosomal evolution [23]. In the *Orchis mascula* group, cytogenetic differences between closely related taxa (*O. mascula* s.l. and *O. provincialis*) are associated with molecular divergence [10,23] and the occurrence of hybrid forms such as *Orchis* × *penzigiana*. All examined individuals of *Orchis* × *penzigiana* show a chromosome number of 2n = 42 and C-banding patterns similar to both parents. The intermediate karyological features of these hybrids further support the role of chromosomal variation in reproductive interactions [27]. More broadly, the contrasting heterochromatin organization between groups such as *Neotinea* and *Orchis* s.s. suggests that shifts in repetitive DNA structure may underlie genomic incompatibility. Such differences can influence chromosome pairing, recombination and gene regulation, thereby acting as potential reproductive barriers.

Taken together, these findings support the hypothesis that heterochromatin dynamics and repetitive DNA evolution are important for genome differentiation in Orchidinae. Changes in heterochromatin distribution not only reflect evolutionary history but may actively contribute to speciation by promoting genetic divergence [115].

In summary, with regard to karyotype symmetry in relation to heterochromatin distribution, analyses conducted in the 32,36-chromosome group (e.g., *Anacamptis*, *Ophrys* and *Serapias*) have provided important insights. For the 40,42-chromosome group, data on constitutive heterochromatin, particularly those obtained for the *Orchis mascula* group, remain significant. However, information for the genus *Dactylorhiza* is still limited. Moreover, the application of FISH techniques remains insufficient for both groups.

### Fluorescence in Situ Hybridization (FISH) Analysis in Selected Species

The 32,36-chromosome species exhibit variation in the number and chromosomal position of 18S-25S and 5S rDNA sites (Table 3). In the genus *Anacamptis*, *A. morio* shows interpopulation variation (e.g., four or six major rDNA sites). Moreover, *A. papilionacea* has been reported to possess either two 18S-25S rDNA and four 5S rDNA sites, or three 18S-25S rDNA and five 5S rDNA sites [19,32]. *Himantoglossum robertianum* presents a unique feature, with the 5S and 18S-25S rDNA signals located on opposite arms of the same chromosome. These rDNA site patterns provide valuable insights into interspecific and intergeneric divergence. In *H. hircinum*, FISH revealed four 18S-5.8S-25S rDNA sites and four 5S rDNA sites [32]. In *Ophrys tenthredinifera*, using the pTa71 (18S-5.8S-25S) probe, FISH detected three hybridization signals, consistent with Ag-NOR staining of interphase nuclei. This species displayed two pairs of 5S rDNA sites [33]. In *Serapias vomeracea*, FISH revealed six 5S rDNA signals, two of which were more intense than the others, and six 18S-25S rDNA signals [33].

In the 40,42-chromosome groups, fewer data are available, but current evidence suggests a more conservative pattern of rDNA site distribution. The lack of FISH analyses of *Orchis* s.s. and *Neotinea* s.l. limits interpretation, although fluorochrome banding indicates consistent organization of repetitive DNA. In *Dactylorhiza*, a combination of genomic in situ hybridization (GISH) and FISH has been applied to *D. majalis* and *D. traunsteineri* [34]. More recently, Naczk et al. [35] reported FISH analyses in diploid *D. incarnata* and *D. fuchsii* (2n = 40), which showed one pair of 5S rDNA loci and either one or two pairs of 35S rDNA loci, all in subterminal positions. Similar analyses were conducted in the polyploids *D. majalis* and *D. maculata* (2n = 80).

Table 3 summarizes the results obtained for selected species within the 32,36- and 40,42-chromosome groups. However, there is a shortage of data generated by both traditional methods (e.g., C-banding with Giemsa and fluorochromes) and FISH. Indeed, few data are reported for AT- and GC-rich repetitive sequences detected using the fluorochromes DAPI, Hoechst and CMA. Furthermore, there is a complete lack of FISH data for the genera *Orchis* s.s., *Neotinea*, *Gymnadenia* and *Platanthera*.

## 6. Karyomorphological Comparison Between Groups

### 6.1. Comparative Karyomorphology in the 32,36-Chromosome Group

Within the 32,36-chromosome group, the genera *Anacamptis*, *Himantoglossum* and *Ophrys* exhibit broadly similar karyomorphology, with chromosomes ranging from 1.45 to 5.31 µm in length [5,12,19,33]. In contrast, the genus *Serapias* shows less distinct centromeric morphology and smaller chromosome sizes, ranging from 1.27 to 3.62 µm [88]. This karyomorphology is highlighted by Giemsa banding: only the *Serapias* group shows large centromeric heterochromatin bands, whereas species in the other genera exhibit only limited heterochromatic regions. In *Anacamptis*, telomeric bands can be observed in *A. papilionacea* and *A. fragrans*, coexisting with bands revealed by Hoechst or DAPI staining [5,19,58] (Table 4).

However, within the 32,36-chromosome group, the genus *Anacamptis*, as studied using traditional methods, includes only two species with chromosomes rich in constitutive heterochromatin (*A. coriophora* and *A. papilionacea*), whereas the other species show little visible heterochromatin. In contrast, the genus *Serapias* stands out within this group for its high constitutive heterochromatin content.

### 6.2. Comparative Karyomorphology in the 40,42-Chromosome Group

Karyological investigations of these chromosome groups have revealed considerable diversity in chromosome number, morphology and heterochromatin content. Species within *Dactylorhiza* and *Gymnadenia* (both 2n = 40) and *Orchis* s.s. (2n = 42) show highly similar karyomorphology, characterized by smaller chromosomes (0.94–3.32 µm) than those of the 32- and 36-chromosome genera, such as *Anacamptis*, *Himantoglossum* and *Serapias* [19].

However, Giemsa banding highlights notable differences in heterochromatin distribution. In *Dactylorhiza romana* and *D. phoenissa*, entirely heterochromatic accessory chromosomes have been identified [20,56,58], in addition to the pronounced centromeric and telomeric bands that are typical of *Dactylorhiza* species and are also seen in some *Gymnadenia* s.l. species.

The *Orchis* s.s. group shows distinct responses to Giemsa banding, fluorochrome Hoechst 33258 and DAPI. The *Orchis mascula* complex (e.g., *O. mascula*, *O. provincialis*, *O. pauciflora*, *O. patens*) exhibits cytogenetic characteristics that differ from those of the *O. militaris* complex (e.g., *O. militaris*, *O. simia*, *O. italica*) [58]. Indeed, in the *O. mascula* group, constitutive heterochromatin distribution is markedly different from that of the *O. militaris* group. Furthermore, cytogenetic analyses indicate a close affinity between *O. mascula* and *O. pauciflora*, but not between *O. mascula* and *O. provincialis* or *O. patens* [20]. The presence of extensive telomeric heterochromatin and broad centromeric C^+^ bands in *O. mascula* suggests species-specific chromosomal restructuring. The absence of Hoechst/DAPI signals in certain species implies differences in repetitive DNA composition, indicating genomic differentiation. These variations may relate to epigenetic processes and reflect functional divergence, potentially linked to adaptation or reproductive isolation [31,33].

Within the 42-chromosome group, the genera *Platanthera* (in the limited species examined) and *Chamorchis* display intermediate karyomorphological features and heterochromatin distribution patterns between those of the 32,36- and 40,42-chromosome groups [33,48]. For example, the chromosomes of some *Platanthera* species are larger than is typical for the 42-chromosome group, with distinct centromeric heterochromatin [33].

With a well-defined centromere position and chromosomes rich in constitutive heterochromatin, the chromosomal morphology of *Chamorchis alpina* is clearly distinct from that of species with chromosome numbers 2n = 40 or 42 [48]. Conversely, *Traunsteinera globosa* exhibits chromosomes similar in size to those of *Neotinea* s.l., with centromeric heterochromatin resembling that of *N. tridentata* [38].

Thus, in the 40,42-chromosome group, many species exhibit a moderate amount of constitutive heterochromatin, particularly those belonging to the *Orchis mascula* complex. Notably, accessory chromosomes have been reported in *Dactylorhiza romana* and *D. phoenissa*; these are entirely heterochromatic and have not been observed in species of the 32,36-chromosome group.

## 7. Epigenetic Factors

Orchids represent a group of plants in which epigenetic phenomena play a substantial role, particularly concerning environmental adaptation, reproduction and symbiotic interactions with microorganisms [31,116,117]. In recent years, cytogenetic studies of genera belonging to the Orchidinae subtribe have highlighted phenomena that may be attributable to epigenetic mechanisms [19,20,31,33]. Karyomorphological analyses of certain species of the genus *Ophrys* (e.g., *O. tardans*, *O. tenthredinifera*) and *Orchis* s.s. (e.g., *O. mascula*) have revealed interesting features potentially linked to epigenetic processes [20,33].

Thus, within the Orchidaceae, epigenetics provides a valuable framework for clarifying species boundaries among morphologically similar taxa that nevertheless display functional and ecological differentiation. It should be emphasized, however, that such variation may result from both genetic mutations and epigenetic modifications, often acting together, with the relative influence of each process depending on the evolutionary history and environmental context of the species involved [118].

## 8. Conclusions

The study of plant biodiversity for the purposes of its conservation [119], especially in biodiversity hotspots such as the Mediterranean region [120], includes research into flora [121,122,123,124], plant communities [125,126,127,128,129], taxonomy (including comparative studies [130,131] and the description of new rare and endangered species [132,133,134]) and population dynamics [135], as well as molecular and cytogenetic analyses [19,20,28,33,75,76,87].

Given its wide variability, the Orchidaceae family represents an interesting group with which to monitor biodiversity at a global level.

Chromosome analyses (e.g., chromosome counts, karyomorphology, Giemsa banding and the use of the fluorochromes DAPI, Hoechst, and CMA3) have proved robust and reliable, particularly in the 32,36-chromosome group. For the 40,42-chromosome group, analyses were more reliable when based on Giemsa banding and the use of the fluorochromes DAPI, Hoechst and CMA3. However, further data are needed for this latter group.

Regarding the cytogenetic diversity of Orchidinae species in the Mediterranean region, two points seem clear. First, our investigation showed wide variability in chromosome number and karyomorphology. Second, observational studies have found that large amounts of constitutive heterochromatin in some groups sometimes play a role in species differentiation.

The 32,36-chromosome orchid group, particularly *Anacamptis* s.l. and closely related genera, in which karyotypic diversity is driven mainly by structural heterochromatin variation, can be seen as a more conserved model.

In contrast, the 40,42-chromosome group, encompassing genera such as *Neotinea*, *Orchis*, *Dactylorhiza* and *Gymnadenia*, displays more advanced karyotype complexity, hybrid origins and polyploid evolution, possibly indicating greater cytogenetic complexity.

These differences in chromosomal architecture, heterochromatin dynamics and polyploid behavior significantly enhance our understanding of speciation and diversification within the Orchidinae.

As summarized in this overview, there have been numerous cytogenetic investigations of Orchidinae species, demonstrating the remarkable karyotypic variability within this subgroup of Orchidaceae and highlighting its suitability for studies of karyotype evolution. Nevertheless, cytogenetic research into Orchidinae is far from complete. In several genera (e.g., *Dactylorhiza*, *Orchis* s.s., *Platanthera*), important questions remain unresolved, and in some groups only a limited number of species have been analyzed, while for others there is still no cytogenetic data at all.

In conclusion, chromosomal variability in the Orchidinae of the Mediterranean region indicates that this lineage has evolved through multiple interacting mechanisms, including chromosomal restructuring, heterochromatin diversification, polyploidy and epigenetic processes, ultimately contributing to the extraordinary morphological and genetic diversity that now characterizes this subtribe.

## Figures and Tables

**Table 1:** Chromosome numbers for each taxon (the genera are indicated in bold).

Taxon	Chromosome Numbers	References
***Anacamptis ****champagneuxii* (Barnéoud) R.M.Bateman, Pridgeon & M.W.Chase	36, 72	[39,40]
*A. collina* (Banks & Sol. ex Russell) R.M.Bateman, Pridgeon & M.W.Chase	36	[5,19,41]
*A. coriophora* (L.) R.M.Bateman, Pridgeon & M.W.Chase	36	[42]
*A. fragrans* (Pollini) R.M.Bateman, Pridgeon & M.W.Chase	36	[5,19,42,43]
*A. laxiflora* (Lam.) R.M.Bateman, Pridgeon & M.W.Chase	36	[5,40,44,45]
*A. longicornu* (Poir.) R.M.Bateman, Pridgeon & M.W.Chase	36	[5]
*A. morio* (L.) R.M.Bateman, Pridgeon & M.W.Chase	36	[5,19,28,33,40,41]
*A. palustris* (Jacq.) R.M.Bateman, Pridgeon & M.W.Chase	36, 36 + 1B	[5,19,41]
*A. papilionacea* (L.) R.M.Bateman, Pridgeon & M.W.Chase	32, 32 + 1B	[5,19,28,41]
*A. pyramidalis* (L.) Rich.	36, 72, 72 + 1B	[19,25,45,46]
***Chamorchis ****alpina* (L.) Rich.	42	[47,48]
***Dactylorhiza ****elata* (Poir.) Soó	80	[49,50]
*D. foliosa* (Verm.) Soó	40	[50,51]
*D. fuchsii* (Druce) Soó	40, 80	[35,50,52,53]
*D. iberica* (M.Bieb. ex Willd.) Soó	40	[50,54]
*D. incarnata* (L.) Soó	40	[34,35,50,52,53]
*D. insularis* (Sommier) Landwehr	60	[42,44]
*D. maculata* (L.) Soó	80	[35,50,52]
*D. maculata* (L.) Soó subsp. *maculata*	80	[55]
*D. majalis* (Rchb.) P.F.Hunt & Summerh	80	[34,35]
*D. majalis* subsp. *praetermissa* (Druce) D.M.Moore & Soó	80	[52]
*D. phoenissa* (B.Baumann & H.Baumann) P.Delforge	80, 80 + 4B	[56]
*D. romana* (Sebast.) Soó	40, 40 + 1 − 3B	[20,57,58]
*D. saccifera* (Brongniart) Soó	40	[39,50,58]
*D. sambucina* (L.) Soó	40	[20,50]
*D. traunsteineri* (Saut. ex Rchb.) Soó	80	[53]
*D. urvilleana* (Steudel) Baumann & Künkele	80	[50]
*D. viridis* (L.) R.M.Bateman, Pridgeon & M.W.Chase	40	[43,59]
***Gennaria ****diphylla* (Link.) Parl.	34	[60,61]
***Gymnadenia ****archiducis-joannis* (Teppner & Klein) Teppner & Klein	80	[62]
*G. buschmanniae* (Teppner & Ster) Teppner & Klein	100	[48,63]
*G. conopsea* (L.) R.Br.	40	[20,48,64]
*G. corneliana* (Beauv.) Teppner & Klein	40	[65]
*G. dolomitensis* Teppner & Klein	80	[66]
*G. gabasiana* (Teppner & Klein) Teppner & Klein	40	[67]
*G. lithopolitanica* (Ravnik) Teppner & Klein	40	[62]
*G. miniata* (Crantz) Hayek	80	[62]
*G. odoratissima* (L.) Rich.	40	[20,48,64]
*G. rhellicani* (Teppner & Klein) Teppner & Klein	40	[20,48,68]
*G. widderi* (Teppner & Klein) Teppner & Klein	80	[62]
***Herminium ****monorchis* (L.) R.Br.	40	[52]
***Himantoglossum ****adriaticum* H.Baumann	36, 36 + 1B	[43,45]
*H. hircinum* (L.) Spreng.	36	[41,43]
*H. robertianum* (Loisel.) P.Delforge	36	[19,42]
***Neotinea ****lactea* (Poir.) R.M.Bateman, Pridgeon & M.W.Chase	42, 42 + 1B	[20,42,44]
*N. maculata* (Desf.) Stearn	42	[20,39]
*N. commutata* (Tod.) R.M.Bateman	84	[20,69,70]
*N. tridentata* (Scop.) R.M.Bateman, Pridgeon & M.W.Chase	42, 42 + 1B	[20,41]
*N. ustulata* (L.) R.M.Bateman, Pridgeon & M.W.Chase	42, 42 + 1B	[20,41]
***Ophrys ****aesculapii* Renz	36	[71]
*O. apifera* Huds.	36	[19,57,72,73,74]
*O. apulica* (O.Danesch & E.Danesch) O.Danesch & E.Danesch	36, 72	[19,28,57,75]
*O. arachnitiformis* Grenier & Philippe	36	[44,59]
*O. archimedea* P.Delforge & M.Walravens	36	[76]
*O. archipelagi* Gölz & Reinhard	36	[77]
*O. aspea* Devillers-Tersch. & Devillers	36	[78]
*O. benacensis* (Reisigl) O.Danesch, E.Danesch & Ehrend.	36	[72]
*O. bertolonii* Mor.	36	[19,74,79]
*O. bertoloniiformis* O.Danesch & E.Danesch	36	[72]
*O. biscutella* O.Danesch & E.Danesch	36	[19,72]
*O. bombyliflora* Link	36	[19,74,79]
*O. caesiella* P.Delforge	36	[74]
*O. candica* (E.Nelson ex Soó) H.Baumann & Künkele	36	[57]
*O. celiensis* (O.Danesch & E.Danesch) P.Delforge	36	[19,74]
*O. chestermanii* (J.J.Wood) Gölz & Reinhard	36	[80]
*O. classica* Devillers-Tersch. & Devillers	36	[19,79]
*O. dyris* Maire	72, 90	[73]
*O. eleonorae* Devillers-Tersch. & Devillers	36	[78]
*O. exaltata* Ten.	36	[59]
*O. ferrum-equinum* Desf.	36	[81]
*O. flammeola* P.Delforge	36	[76]
*O. forestieri* (Rchb.f.) Lojac.	36, 72	[74,75]
*O. funerea* Viv.	36	[76]
*O. fusca* L.	36, 72	[19,44,72,74,79]
*O. garganica* O.Danesch & E.Danesch	36	[19,73]
*O. gazella* Devillers-Tersch. & Devillers	36	[78]
*O. holosericea* (Burm.f.) Greuter	36	[72]
*O. incubacea* Bianca	36	[19,79]
*O. insectifera* L.	36	[19,72,74]
*O. iricolor* Desf.	36	[74]
*O. lacaitae* Lojac.	36	[19,69,74]
*O. laurensis*	36	[76]
*O. lojaconoi*	36	[76]
*O. lucifera* P.Delforge, Devillers-Tersch. & Devillers	36	[76]
*O. lunulata* Parl.	36	[59,74]
*O. lutea* Cav.	36	[19,44,73,74,79]
*O. lycia* Renz & Taubenheim	36	[82]
*O. mammosa* Desf.	36	[82]
*O. mateolana* Medagli, D’Emerico, Bianco & Ruggiero	36	[19,77]
*O. morisii* (U.Martelli) Soó	36	[75]
*O. obaesa* Lojac.	36	[76]
*O. oestrifera* M.Bieb.	36	[81]
*O. omegaifera* H.Fleischm	36	[82]
*O. oxyrrhynchos* Tod.	36	[69]
*O. pallida* Raf.	36	[19,69,76]
*O. parvimaculata* (O.Danesch & E.Danesch) Paulus & Gack	36	[19,57,75]
*O. peucetiae* Lozito, D’Emerico, Medagli & Turco	36	[75]
*O. praecox* (Corrias) Devillers-Tersch. & Devillers	36	[74]
*O. promontorii* O.Danesch & E.Danesch	36	[72]
*O. pseudomelena* Turco, Medagli & D’Emerico	36	[75]
*O. reinholdii* Spruner ex H.Fleischm.	36	[82]
*O. scolopax* Cav.	36, 36 + 1B	[72,73,74]
*O. sicula* Tineo	36	[19,74,79]
*O. sipontensis* Lorenz & Gembardt	36	[72]
*O. speculum* Link	36	[44]
*O. tardans* O.Danesch & E.Danesch	36	[19,28,74]
*O. tarentina* Gölz & Reinhard	36	[19,57,74]
*O. tenthredinifera* Willd. s.l.	36	[33,72,74]
*O. umbilicata* Desf.	36	[19]
*O. vasconica* (O.Danesch & E.Danesch) P.Delforge	72	[73]
***Orchis ****anatolica* Boiss.	42	[51]
*O. anthropophora* (L.) All.	42	[19,44]
*O. brancifortii* Biv.-Bern.	42	[69]
*O. ichnusae* (Corrias) Devillers-Tersch. & Devillers	42	[20]
*O. italica* Poir.	42	[25]
*O. mascula* (L.) L.	42, 42 + 1B	[20,27,83]
*O. militaris* L.	42	[54]
*O. olbiensis* Reut. ex Gren.	42	[84]
*O. patens* Desf.	84	[85]
*O. pauciflora* Ten.	42	[84,85,86]
*O. provincialis* Balb. ex Lam. & DC.	42	[20,27,44,85]
*O. purpurea* Huds.	42	[20,54]
*O. quadripunctata* Cirillo ex Ten.	42	[80]
*O. simia* Lam.	42	[54]
*O. spitzeli* Sauter	42	[83]
***Platanthera ****algeriensis* Batt. & Trab.	42	[87]
*P. bifolia* (L.) Rich.	42	[52,64]
*P. chlorantha* (Custer) Rchb.	42	[52,64,87]
***Pseudorchis ****albida* (L.) A.Löve & D.Löve	42	[64]
***Serapias ****apulica* (Baumann & Künkele) P.Delforge	36	[41,88]
*S. bergonii* E.G.Camus	36	[19,42,88]
*S. cordigera* L.	36	[19,64,88]
*S. gregaria* Godfery	72	[89]
*S. lingua* L.	72	[19,64,88]
*S. neglecta* De Not.	36	[59]
*S. nurrica* Corrias	36	[88,90]
*S. olbia* Verguin	72	[89]
*S. orientalis* (Greuter) H.Baumann & Künkele	36	[19,41]
*S. parviflora* Parl.	36	[19,54,88]
*S. perez-chiscanoi* Acedo	36	[73,89]
*S. politisii* Renz	36	[88,91]
*S. strictiflora* Welwitsch ex Veiga	72	[89]
*S. vomeracea* (Burm.f.) Briq.	36	[42,88]
***Traunsteinera ****globosa* (L.) Rchb.	42	[38,54,92]

**Table 2:** List of diploid genera with chromosome numbers (2n).

Subtribe	Genus	Chromosome Numbers (2n)
*Orchidinae*	*Gennaria*	34
	*Anacamptis* s.l.	32, 36
	*Himantoglossum*	36
	*Ophrys*	36
	*Serapias*	36
	*Dactylorhiza* s.l.	40
	*Gymnadenia* s.l.	40
	*Herminium*	40
	*Chamorchis*	42
	*Neotinea* s.l.	42
	*Orchis* s.s.	42
	*Platanthera*	42
	*Pseudorchis*	42
	*Traunsteinera*	42

**Table 3:** Reported chromosome numbers, AT-rich locations (4′-6-diamidino-2-phenyl-indole (DAPI)/Hoechst 33258), GC-rich locations (chromomycin A3 (CMA)) and the number of sites with 25S rDNA and 5S rDNA in Orchidinae species (adapted from Ref. [38]). Abbreviations: tb, telomeric band; cb, centromeric band; stb, subtelomeric band.

Taxa	Chromosome Number (2n)	DAPI/ Hoechst 33258 (AT-rich)	CMA3 (GC-rich)	Number of Sites with 18S-5.8S-25S rDNA	Number of Sites with 5S rDNA
*Anacamptis collina*	36			2 or 3	2
*A. coriophora*	36	tb			
*A. morio*	36			2 or 6	2 or 4
*A. papilionacea*	32	tb		2 or 3	4 or 5
*Himantoglossum robertianum*	36			1	1
*H. hircinum*	36			4	4
*Ophrys fusca*	36		cb		
*O. incubacea*	36		cb and tb		
*O. tenthredinifera*	36		cb	3	4
*Serapias vomeracea*	36			6	6
*Dactylorhiza fuchsii* [35]	40			3	2
*D. incarnata* [35]	40			2	2
*D. romana*	40	tb			
*D. saccifera*	40	tb			
*Neotinea lactea*	42	cb and tb			
*N. tridentata*	42	cb			
*Orchis mascula*	42	tb and stb			
*O. provincialis*	42	tb and stb			
*Platanthera chlorantha*	42	cb			

**Table 4:** Main cytogenetic comparisons between 32,36-chromosome and 40,42-chromosome orchid groups.

Feature	32,36-Chromosome Group (e.g., *Anacamptis*, *Himantoglossum*, *Ophrys, Serapias*)	40,42-Chromosome Group (e.g., *Dactylorhiza*, *Gymnadenia*, *Neotinea*, *Orchis*)
Chromosome number	Mostly 2n = 36 (some cytotypes 2n = 34, 52, 54, 72).	Mostly 2n = 40 or 42 (polyploids: 2n = 60, 63, 80, 84, 100, 120).
Karyotype symmetry	Variable: symmetrical (e.g., *A. morio*, *A. collina*), asymmetrical (e.g., *A. papilionacea*, *A. coriophora*).	Often more symmetrical (e.g., *Neotinea tridentata*, *Orchis mascula*).
Heterochromatin patterns	Centromeric and subtelomeric; varies with asymmetry. Large centromeric bands in all *Serapias* species; telomeric bands in *Anacamptis papilionacea*, *A. coriophora* and *A. fragrans*.	Strong centromeric heterochromatin (e.g., *Orchis mascula*). DAPI+/Hoechst+ telomeric regions in *Neotinea* and *Orchis*.
Polyploidy & hybridization	Frequent: auto- and allopolyploids observed (e.g., *Anacamptis pyramidalis*, *A.* × *gennarii*, *Serapias lingua*, *Ophrys tenthredinifera*).	Complex: clear examples of both auto- and allopolyploidization (e.g., *Neotinea commutata*, *Orchis italica*).
Meiotic behavior	Presence of univalents, bivalents and trivalents in triploids and tetraploids (e.g., *A. laxiflora*, *A.* × *gennarii*, *S. lingua*).	Stable bivalent formation in allopolyploids (e.g., *Neotinea commutata*).
rDNA site number (FISH)	High variation (e.g., 2–6 18S-25S sites, 2–5 5S sites). e.g., *A. morio*, *S. vomeracea*, *H. robertianum*.	FISH not widely applied (e.g., not in *Neotinea* s.l., *Orchis* s.s.). FISH reported in *Dactylorhiza maialis*, *D. traunsteineri D. incarnata*.
B-chromosomes	Reported in *Anacamptis papilionacea*, 2n = 32 + 1B; *Anacamptis palustris*, 2n = 36 + 1B; and *Ophrys*, 2n = 36 + 1B.	Observed in *Dactylorhiza romana*, 2n = 40 + 1 − 3B; *D. phoenissa*, 2n = 80 + 4B; *Neotinea lactea* and *Orchis mascula*, 2n = 42 + 1B.
Karyotype evolution	Suggests more ancestral status. However, some genera (e.g., *Serapias*) have extensive chromosomal rearrangements.	Suggests polyploidy-driven evolutionary transformation and greater structural complexity.
Banding techniques	Giemsa, DAPI, Hoechst show correlation with karyotype symmetry. G-C content variable.	Strong correspondence between DAPI/Hoechst and C-banding (e.g., *Orchis lactea*). Epigenetic heterochromatin modifications (e.g., *Orchis mascula*, *O. pauciflora*).
Reproductive isolation	No known intergeneric hybrids with *Ophrys*. Karyotype differentiation may act as barrier.	Hybridization common within genera. Intergroup hybridization seen (e.g., *O. mascula* × *O. provincialis*).
Phylogenetic status	Probably more ancestral (*Anacamptis*, *Himantoglossum*, *Ophrys*), having conserved karyotypes.	Probably more evolved, with polyploid complexity.

## Data Availability

Data are contained in the paper.
